# Karyotypic variation in the long-whiskered catfish *Pimelodusblochii* Valenciennes, 1840 (Siluriformes, Pimelodidae) from the lower Tapajós, Amazonas and Trombetas Rivers

**DOI:** 10.3897/CompCytogen.v12i3.22590

**Published:** 2018-08-02

**Authors:** Ivanny Coelho da Fonseca, Luan Aércio Melo Maciel, Frank Raynner Vasconcelos Ribeiro, Luís Reginaldo Ribeiro Rodrigues

**Affiliations:** 1 Graduate Program in Amazonian Natural Resources – PPGRNA, Federal University of Western Pará – UFOPA, Av. Mendonça Furtado, 2°Andar, Nº 2440, Bairro Aparecida CEP: 68040-050, Santarém, PA, Brazil Federal University of Western Pará Santarém Brazil; 2 Graduate Program in BioSciences – PPGBio, Federal University of Western Pará – UFOPA, Av. Mendonça Furtado, 2°Andar, Nº 2440, Bairro Aparecida CEP: 68040-050, Santarém, PA, Brazil Federal University of Western Pará Santarém Brazil; 3 Water Science and Technology Institute – ICTA, Federal University of Western Pará, Campus Amazonia, Av. Mendonça Furtado, 2°Andar, Nº 2946, Bairro Fátima CEP: 68040-050, Santarém, PA, Brazil Federal University of Western Pará Santarém Brazil; 4 Genetics and Biodiversity Laboratory – LGBio, Federal University of Western Pará – UFOPA, Rua Vera Paz, s/n, Salé, 68035-110, Santarém, PA, Brazil Federal University of Western Pará Santarém Brazil

**Keywords:** Amazon basin, catfish, *
Pimelodus
*, rDNA, species complex

## Abstract

The genus *Pimelodus* LaCépède, 1803 comprises 35 formally recognized species distributed along the major neotropical river basins. Despite conservatism in diploid number with 2n=56, an intense variation of chromosomal morphology (karyotypic formula) has been documented in *Pimelodus* species. In the present study, we analyzed karyotypes of 20 specimens, identified as *Pimelodusblochii* Valenciennes, 1840 and collected from the lower courses of the Tapajós, Amazonas and Trombetas Rivers. The karyotypes were characterized by Giemsa conventional staining, C-banding, silver staining (Ag-NOR) and fluorescent in situ hybridization (FISH) with 5S and 18S rDNA probes. The karyotypes showed 2n=56 chromosomes in fish from the Tapajós River. In contrast, fish from the Amazonas and Trombetas Rivers had 2n=58. The nucleolus organizing regions were labeled on the short arm of an acrocentric chromosome as demonstrated by silver staining and FISH. Signals for 18S and 5S rDNA were co-localized on one chromosome pair. Our results demonstrate karyotypic divergence between Tapajós and Amazonas-Trombetas populations of *P.blochii*, interpreted as supporting the existence of a species complex in this taxon.

## Introduction

The genus *Pimelodus* LaCépède, 1803 (Siluriformes, Pimelodidae) comprises 35 valid species exclusively distributed in neotropical freshwater drainages. It is commonly recorded in the Amazonas, Orinoco, Araguaia-Tocantins, São Francisco, and Paraná-Paraguay River basins. In the Amazon basin, seven species of *Pimelodus* have been recorded: *Pimelodusalbofasciatus* Mees, 1974, *Pimelodusblochii* Valenciennes, 1840, *Pimelodusaltissimus* Eigenmann & Pearson, 1942, *Pimelodusjivaro* Eigenmann & Pearson, 1942, *Pimelodusornatus* Kner, 1858, *Pimeloduspictus* Steidachner, 1876, and *Pimelodustetramerus* Ribeiro & Lucena, 2006 (Ferraris 2007, Eschmeyer and Fong 2017).

Cytogenetic analysis of 32 Pimelodidae family members revealed a conservative karyotypic macrostructure with 2n=56 chromosomes save for a few exceptional karyotypes (2n=50) in *Calophysusmacropterus* Lichtenstein, 1819, *Pinirampuspirinampu* Spix & Agassiz, 1829 and *Luciopimelodusplati* Valenciennes, 1835 species (Ramirez-Gil et al. 1998; Sánchez et al. 2000; Vasconcelos and Santos 2000). Diploid chromosome number variations were also reported in *Pimelodusfur* Lütken, 1874 and *Megalonemaplatanum* Günther, 1880 species samples with 2n=54 and in *Pimelodusblochii* (Della-Rosa et al. 1980) species samples with 2n=58 (Sánchez et al. 2000; Garcia and Moreira-Filho 2008; Carvalho et al. 2011).

Diploid chromosome numbers of eleven previously investigated *Pimelodus* species showed variation from 54 to 58 with eleven distinct karyotype formula (see Table 2 in the Discussion section). Additionally, karyotype variation with B chromosomes had been reported in *P.ortmanni* and *Pimelodus* sp. (Borin and Santos 2004).

So far, two distinct karyotypes were reported for two *Pimelodusblochii* populations; 2n=56 for the Araguaia River, and 2n=58 for the Amazon River (Swarça et al. 2007). Although the chromosome numbers of *P.blochii* were declared in three meetings and published in abstracts by Swarça et al. (2007), no karyotype image of the species is available in any peer-reviewed literature. Therefore, the taxon has been considered as poorly described in the scientific literature.

Rocha (2006) suggested that the name *P.blochii* is arbitrarily assigned to many different long-whiskered catfish in the Brazilian part of the Amazon Basin. Based on morphometric and molecular data, a large collection of *P.blochii* specimens was examined by Rocha (2006). It was demonstrated that the Brazilian specimens are distinct from *P.blochii* topotypes from Suriname and possibly represent a species complex with six undescribed taxa.

In the present paper, we investigate the karyotype of *Pimelodusblochii* from the lower portions of the Tapajós, Amazonas and Trombetas Rivers in order to evaluate their chromosomal features and contribute the debate on the species taxonomy. The karyotypes were characterized by conventional Giemsa staining, C-banding, silver staining (Ag-NOR) and fluorescent in situ hybridization (FISH) technique with 5S and 18S rDNA probes.

## Material and methods

### Samples and collection sites

**Figure 1.**
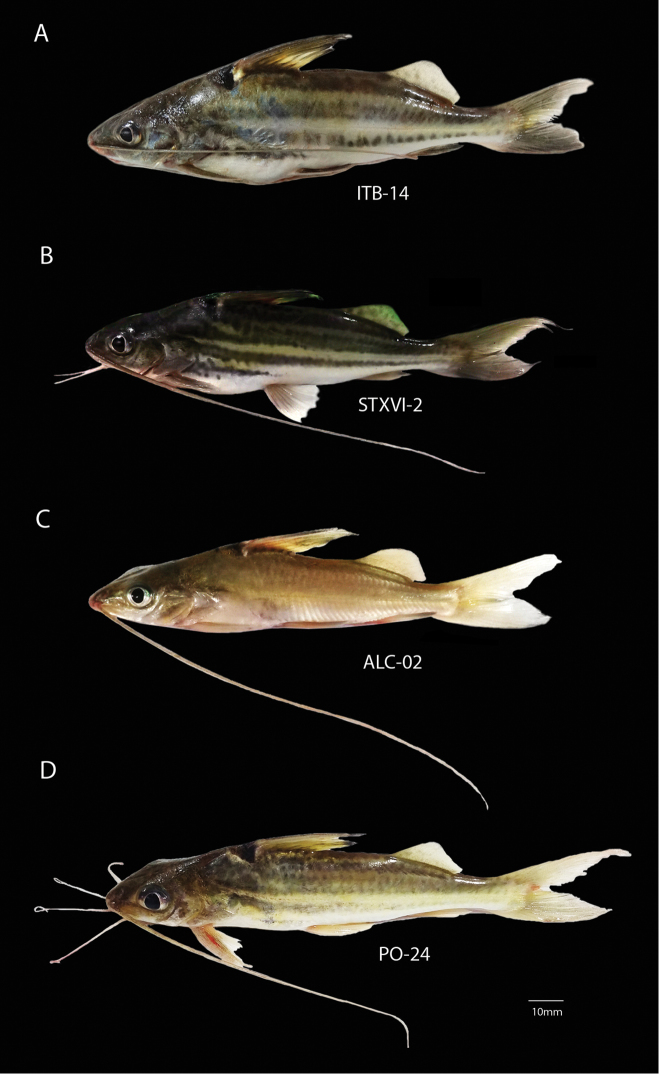
External morphology and coloration of *Pimelodusblochii* specimens examined in the present study. **A** specimen from the Tapajós River (ITB-14, SL=150 mm; W=55 g) **B** specimen from the lower Amazonas River at Santarém (STXVI-2, SL=145 mm; W=52 g) **C** specimen from the lower Amazonas River at Almeirim (ALC-2, SL=110 mm; W=9 g) **D** specimen from the Trombetas River (PO-22, SL=108 mm, W=33 g).

Twenty (20) specimens were collected from four localities in the Tapajós, Amazonas and Trombetas Rivers (Table 1). The fish were captured by local fishermen using hooks and gillnets. The specimens were transferred to plastic tanks (50 L capacity) filled with water from the collection site and aerated with an aquarium pump. After cytogenetic procedures, the specimens were photographed, fixed in 10% formalin for 48 h, washed with running water and preserved with 70% ethanol. The voucher specimens were deposited in the Fish Collection of the Water Science and Technology Institute at Federal University of Western Pará, Brazil. External morphology and coloration features are shown in Fig. 1. The experimental procedures were approved by the Ethical Committee of Animal Research at Federal University of Western Pará (CEUA/UFOPA) under Protocol N. 10001/2015.

**Table 1.** Samples and collection sites of *Pimelodusblochii* in the Amazon Basin.

River	Collection sites	GPS Coordinates (datum WGS84)	n
Tapajós	Itaituba	4°16'12.6"S, 55°58'37.1"W	6
Amazonas	Santarém	2°25'8.0"S, 54°44'28.6"W	6
	Chicaia River, Almeirim	1°38'15.6"S, 52°57'46.2"W	4
Trombetas	Oriximiná	1°45'52.2"S, 55°52'18.8"W	4

**Specimens**: PO-22, PO-25, PO-27, PO-28, ITB-11, ITB14-18, STXVI-1, STXVI-2, STXVI-9, ALC-1-4, PML-5-7.

### Chromosome preparation

Intra-abdominal colchicine (0.0125%) injection was performed at 0.01 ml/g (Bertollo et al. 1978) to stop cell division. The exposed fish were placed in an aerated tank for 40 min and euthanized with water that contains a lethal concentration of clove oil. The posterior kidney tissue was removed and minced in 6 ml of hypotonic KCl solution (0.075 M, 5.6 g/L). The cell suspension was incubated at 37 °C for 20 min and then fixed with fresh methanol-acetic acid (3:1 v/v) solution; the fixative was changed three times.

### Chromosome staining, banding and FISH

Conventional staining was performed with 5% Giemsa solution (phosphate buffer, pH 6.8). The C-banding protocol from Sumner (1972) was followed with minor changes. The NORs were stained with silver nitrate following the Howell and Black (1980) technique.

FISH was used for mapping 18S and 5S rDNA loci (Pinkel et al. 1986). Double FISH experiments were processed with probes generated with 18Sf (5’ CCG CTT TGG TGA CTC TTG AT 3’) and 18Sr (5’ CCG AGG ACC TCA CTA AAC CA 3’) primers, as well as 5Sa (5-TAC GCC CGA TCT CGT CCG ATC) and 5Sb (5-CAG GCT GGT ATG GCC GTA AGC-3) PCR primers (Martins and Galetti 1999; Martins and Vicari 2012).

The PCR products were labeled by nick translation with biotin-14-dATP (BioNick Labeling System kit, Invitrogen/ThermoScientific, Waltham, Massachusetts, USA) and digoxigenin-11-dUTP (DIG-nick translation mix, Roche, Basel, Switzerland). The slides were treated with RNase solution (5 µl RNase 10 mg/mL diluted in 975 µl 2×SSC) for a short period of time. The fixed chromosomes were denatured in 70% formamide (pH 7.0 2×SSC) and heated at 70 °C for 5 min. The hybridization solution mixture was prepared with 20 µl formamide + 8 µl of 50% dextran sulfate + 4 µl of each probe + 4 µl of 20×SSC. The slides were incubated in 2×SSC solution in a humidified and heated (37 °C) chamber overnight.

Post-hybridization washes were performed with 15% formamide at 42 °C for 10 min, three washes in 0.1×SSC at 60 °C for 5 min, and 0.5% Tween20 at room temperature for 5 min. For signal detection, slides were placed in NFDM buffer (20 ml of 20×SSC, pH 7.0 + 5 g of powdered skim milk + 80 ml of distilled water) for 15 min, followed by two washes in 5% Tween20 for 5 min at room temperature.

The hybridized probes were applied in a mixture containing 20 µl anti-digoxigenin-rhodamine (1:200) (Roche, Basel, Switzerland) + 4 µl FITC-Avidin (1:100) (Sigma, St. Louis, Missouri, USA) + 26 µl of C buffer (0.1 M sodium bicarbonate, 0.15 M sodium chloride; pH 7.0) for 60 min. The slides were coated with anti-fading reagent Vectashield H-1000 (Vector Laboratories, Burlingame, California, USA) and chromosomes were counterstained with DAPI (1,2-diamidin-phenyl-indol).

### Microscopy and karyotype analysis

At least 30 metaphases were counted to determine the diploid chromosome number. The best spread metaphase plates were photographed with a CCD camera (Moticam 10 MP) coupled to a Zeiss Axioskop40 microscope for conventional/banding images, and a Nikon Eclipse CI for FISH images. The contrast and brightness were adjusted with ADOBE PHOTOSHOP CS3. The chromosomes were arranged as metacentric (m), submetacentric (sm), subtelocentric (st) and acrocentric (t) following Levan et al. (1964).

## Results

**Figure 2.**
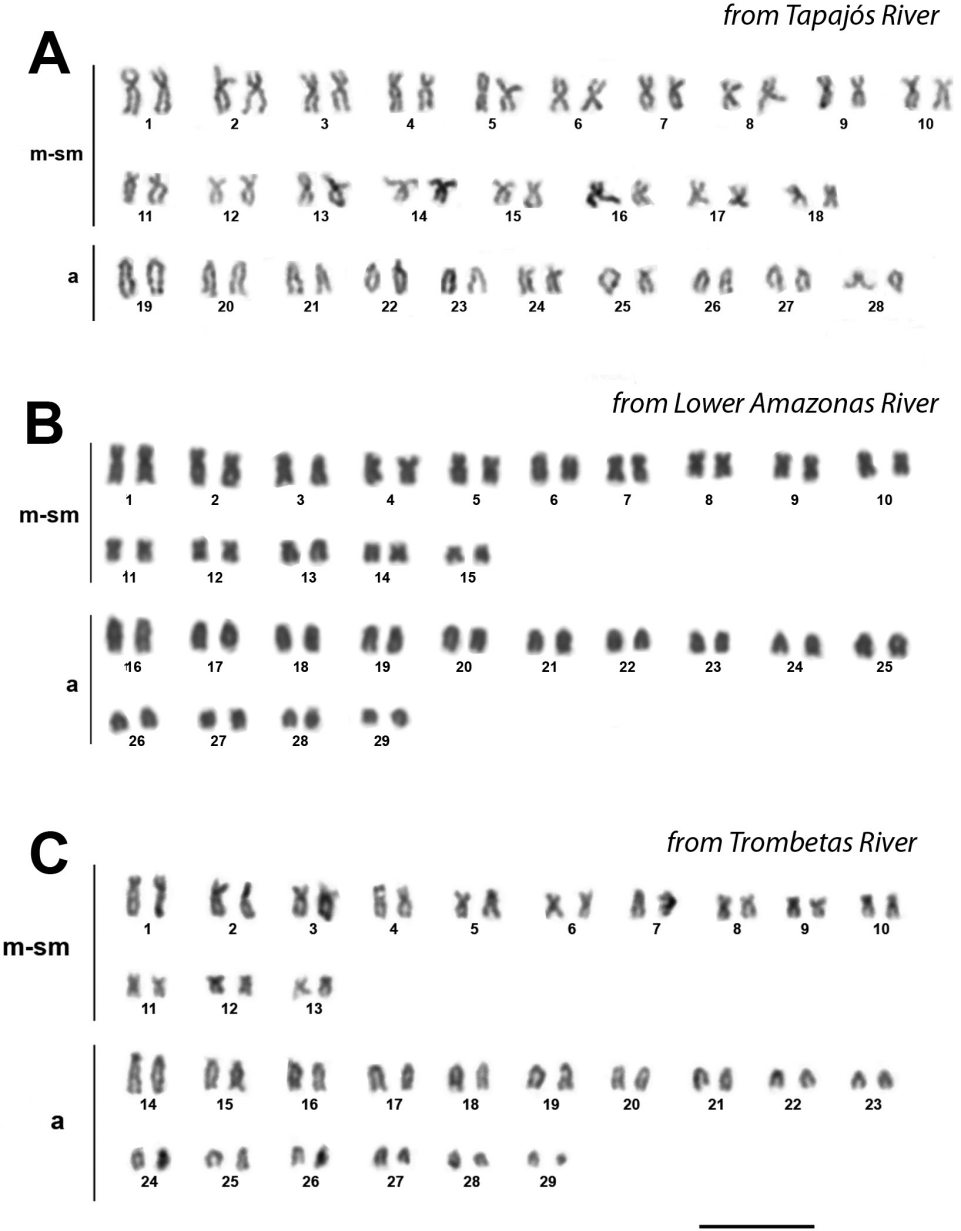
Giemsa-stained karyotypes of *Pimelodusblochii* from the Amazon Basin. **A** 2n=56 chromosomes (36m/sm + 20a) **B** 2n=58 chromosomes (30m/sm + 28a) **C** 2n=58 chromosomes (26m/sm + 32a). Scale bar: 10 μm.

The diploid chromosome number was observed as 56 in the Tapajós River fish (Itaituba population). On the other hand, 2n=58 chromosomes were recorded from Amazonas (Santarém and Almeirim populations) and Trombetas Rivers (Oriximiná population) samples, with minor variation in the karyotypic formula, as 30m/sm+28a and 26m/sm+32a, respectively (Fig. 2).

**Figure 3.**
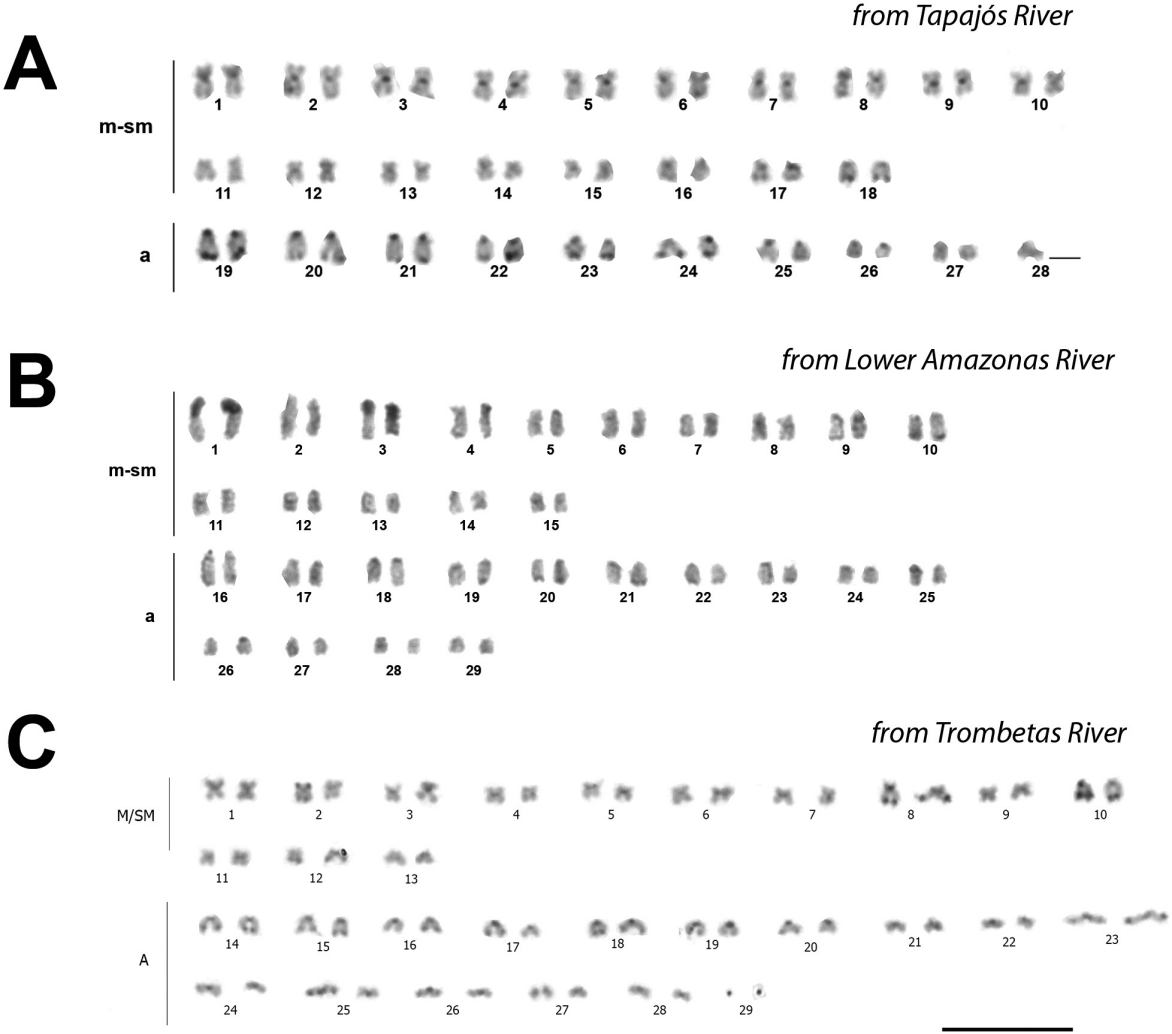
C-banded karyotypes of *Pimelodusblochii* from the Amazon River basin. Scale bar: 10 μm.

**Figure 4.**
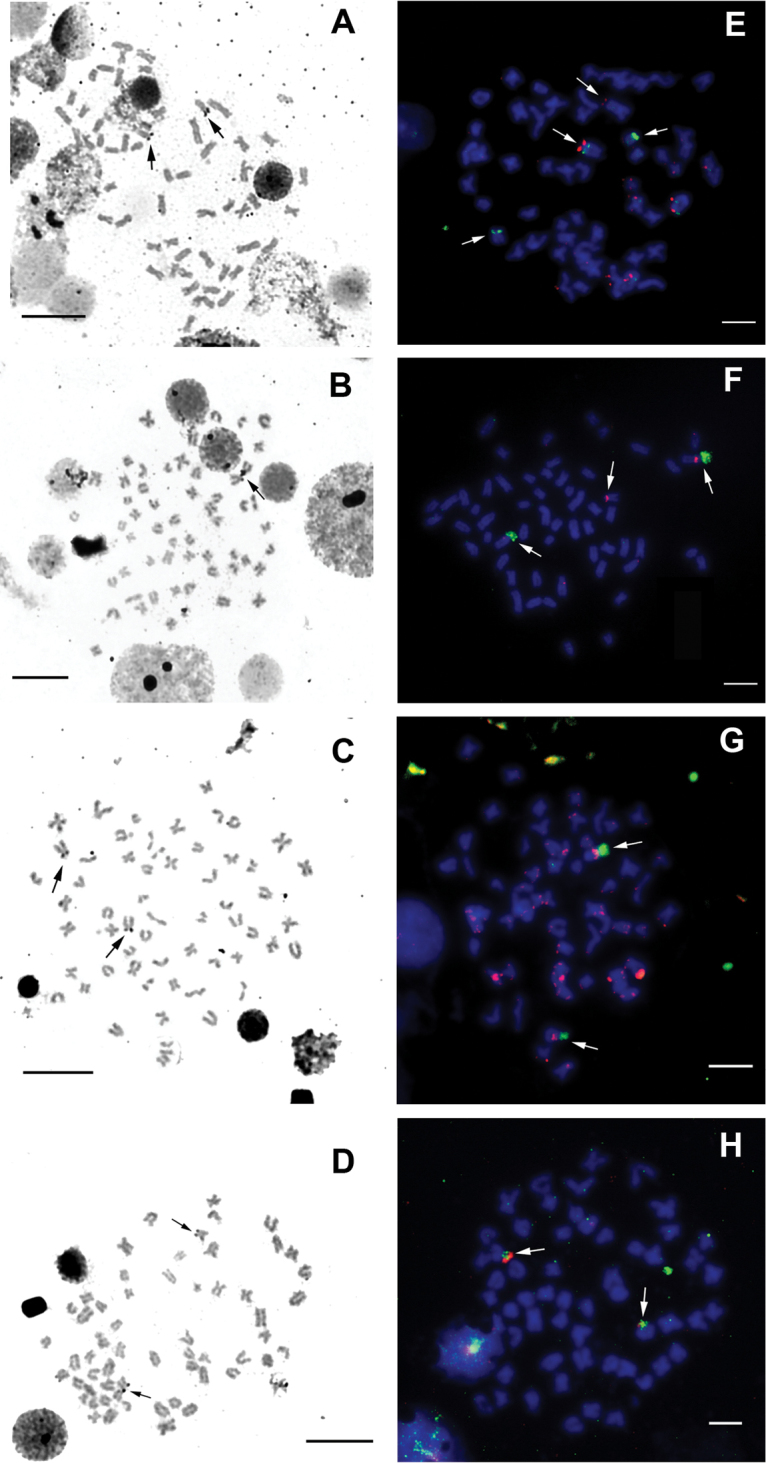
Metaphases of *Pimelodusblochii* showing NOR by silver staining (left) and double FISH of 18S rDNA (green) and 5S rDNA (red) (right). Specimens from the Tapajós River (**A, E**); from the Lower Amazonas River at Santarém (**B, F**), at Almeirim (**D, H**); and from the Trombetas River (**C, G**). Scale bar: 10 μm.

The C-banding results showed small amounts of constitutive heterochromatin in the centromeres. Terminal C-bands were observed in 18 to 24 chromosome pairs from the Tapajós River specimens (Fig. 3). A single NOR-bearing chromosome pair was detected by silver staining in all samples and labeled as the centromeric position (Fig. 4a–d). These sites were compatible with the 18S rDNA locus as demonstrated by FISH (Fig. 4e–h).

**Figure 5.**
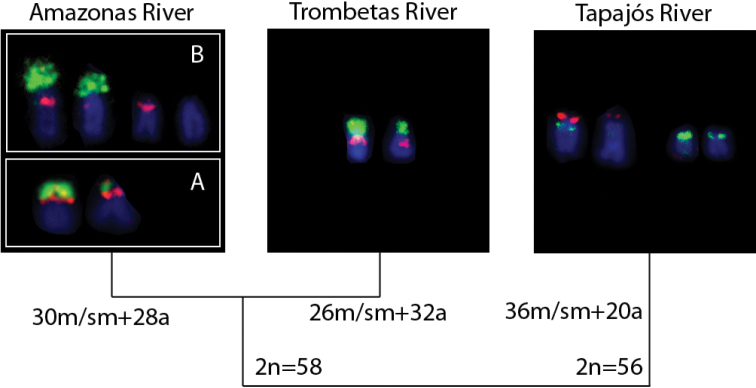
Variation of 18S rDNA (green signal) and 5S rDNA (red signal) chromosomal sites among *Pimelodusblochii* populations from Amazon basin. The samples from Amazonas River showed two distinct patterns: one observed in the Almeirim population (**A**) and other observed in the Santarém population (**B**).

The 5S rDNA probe showed distinct localizations among the samples. Co-localization of 5S and 18S rDNA to a single chromosome pair was detected in the Trombetas and Amazonas Rivers populations (Almeirim population); this syntenic pattern also occurred in the Santarém population but on just one homologous chromosome. The Tapajós River specimens’ karyotypes showed a distinct position for 18S and 5S rDNA (Figs 4, 5).

## Discussion

The karyotype macrostructure of *Pimelodusblochii* from the Tapajós, Amazonas and Trombetas Rivers are compatible with a previous report (Swarça et al. 2007) (Table 2). The diploid chromosome number was found to be 58 (30m/sm+28a) in the lower Amazonas population; this is compatible with the Solimões River population (Della-Rosa et al. 1980). The specimens from the Trombetas River conserved 2n=58 but shifted the karyotypic formula to 26m/sm+32a; this could be a result of pericentric inversions. On the other hand, *Pimelodusblochii* from the Tapajós River with 2n=56 (36m/sm+20a) showed a resemblance to the population from the Araguaia River (Barra do Garça, Mato Grosso State) (Farias et al. 2000).

**Table 2.** Compiled data from the literature on karyotypic traits in *Pimelodus* species. m=metacentric, sm=submetacentric, st=subtelocentric, a=acrocentric, q=long arm, t=terminal, c=centromeric, inter=interstitial, peri=pericentromeric.

Species	2n	Karyotypic formula	18S	5S	References
* Pimelodus fur *	54	32m+8sm+6st+8a	q, t, sm	q inter m, q peri sm	5
* P. microstoma *	56	22m+22sm+6st+6a	q, t, st	peri sm, q peri st	1; 2
* P. argenteus *	56	24m+16sm+12st+4a			3
* P. britskii *	56	24m+18sm+8st+6a	q, t, st	p inter sm, q t st	4
* P. maculatus *	56	32m+12sm+12st	q, t, sm	q inter m, q t sm q peri sm	5; 6
* P. absconditus *	56	24m+18sm+8st+6a	–	–	7
* P. mysteriosus *	56	26m+20sm+2st+8a	–	–	3; 8
* P. ornatus *	56	18m+22sm+6st+10a	–	–	9; 7
* P. ortmanni *	56	24m+18sm+8st+6a	–	–	10; 11; 12
* P. paranaensis *	56	22m+22sm+4st+8a	–	–	13
* P. blochii *	56/58	36m/sm+20st/a; 30m/sm+28a; 26m/sm+32a	q, c, a	q, c, a	14; 15; 16; 17

**References**: 1) Fenocchio et al. (1994); 2) Souza et al. (2004); 3) Souza et al. (2003); 4) Neto et al. (2011); 5) Garcia and Filho (2008); 6) Mazzuchelli et al. (2007); 7) Borin and Santos (2002); 8) Girardi (2015); 9) Abucarma and Santos (1996); 10) Borin and Santos (2004); 11) Terencio et al. (2001); 12) Margarido and Gavasso (2000); 13) Treco and Dias (2009); 14) Della-Rosa et al. (1980); 15) Farias et al. (2000); 16) Silva et al. (2004); 17 (present study).

Despite extensive conservatism in diploid number, variation in karyotypic formula has been frequently detected (Swarça et al. 2007). Pericentric inversions can explain such modifications of chromosomal morphology without alteration of the diploid number and have been previously demonstrated, such as in *Pimelodusmaculatus* and *Pimelodus* sp. (Dias and Foresti 1993).

A single NOR (one pair) is the most common pattern observed in the *Pimelodus* karyotypes (Swarça et al. 2007). This pattern was confirmed for *P.blochii* in the present study. The Ag-NOR sites were coincident with the rDNA 18S FISH signal and showed co-localization with the 5S, a rare condition previously observed in *Pimelodusbritskii* (Neto et al. 2011). In the Order Siluriformes, the synteny of 18S and 5S rDNA cistrons has been reported for *Pimelodus* (Neto et al. 2011; present study), *Imparfinis* Eigenmann et Norris 1900 (Ferreira et al. 2014), *Ancistrus* Kner 1854 (Favarato et al. 2016), *Hemibagrus* Bleeker 1862 (Supiwong et al. 2014), *Corydoras* LaCepède 1803 (Rocha et al. 2016), *Panaqolus* Isbrücker et Schraml 2001 (Ayres-Alves et al. 2017) and *Bunocephalus* Kner 1855 (Ferreira et al. 2017). This syntenic arrangement is interpreted as being less adaptive since in eukaryotes the 45S rRNA genes are transcribed by RNA polymerase I, whereas the 5S are transcribed by RNA polymerase III. This means both processes occur in separate nuclear territories (Amarasinghe and Carlson 1998). Additionally, a linked configuration of 18S and 5S rDNA arrays could favor an undesired disruption of both tandem repeats by means of unequal crossing-over (Martins and Wasko 2004).

Eigenmann (1912) recognized varieties (A and B) of *P.blochii* from Guyana. The A variety has an ashy body pigmentation without dots or stripes, whereas the B variety exhibits four lateral body stripes with the fourth stripe possibly absent or fragmented into dots. *Pimelodusalbofasciatus* (Mees 1974) maintains a close resemblance to the B variety but is distinguished based on eye morphology and dorsal spine length (Ribeiro and Lucena 2006).

Our specimens collected from the Chicaia River, a tributary of the lower Amazonas (Almeirim population), have the typical pigmentation for the A variety, whereas the specimens from the Santarém population, collected at the confluence of the Amazonas and Tapajós Rivers, had the B variety pigmentation. Although both populations conserved the diploid number 2n=58 and karyotypic formula (30m/sm+28a), they diverged in their 18S and 5S rDNA locations (Fig. 5). The specimens from the Trombetas River also have the B variety pigmentation, but diverge in the karyotypic formula (26m/sm+32a) and 18S and 5S rDNA locations (Fig. 5). The most differentiated karyotype was observed in specimens from the Tapajós population; these had a clearly distinct diploid number, karyotypic formula and 18S and 5S location. In general, their coloration resembles the B variety, but their karyotypic distinctiveness leads us to suggest that these specimens may be a new, undescribed *Pimelodus* species.

Ribeiro and Lucena (2006), Azpelicueta et al. (2008), and Lucinda et al. (2016) discussed three distinct patterns of pigmentation among species of *Pimelodus*. According to Lucinda et al. (2016), among the species formally described, a striped-pattern is shared by *P.albicans*, *P.albofasciatus*, and *P.tetramerus*. However, our results, as well as those of Ribeiro and Lucena (2006), suggest that the diversity of *Pimelodus* with blackish stripes along its flanks that inhabit the waters of the Amazonas River basin is greater than the diversity currently described. This includes undescribed species that have commonly been misidentified as *P.blochii*. *P.blochii* from the lower Amazonas River occurs as two morphotypes (A and B) distinguished through body pigmentation and characterized by a 2n=58 karyotype with minor variations. Additional studies of this group are needed in order to clarify the evolutionary dynamics of the 18S and 5S rRNA genes as well as to acquire morphological and molecular data to evaluate the taxonomy and phylogeny of *Pimelodus* species.

## Conclusions

The populations of *Pimelodusblochii* from the lower courses of Amazonas, Tapajós and Trombetas rivers presented differentiated karyotypes based on variation in diploid number and chromosome morphology. The specimens collected from the Tapajós River, with 2n=56, are clearly distinguished from the others and may constitute a new, undescribed *Pimelodus* species.
